# Exciton–Phonon Interactions in Strained Domes of Monolayer MoS_2_ Studied by Resonance Raman Spectroscopy

**DOI:** 10.3390/nano13192722

**Published:** 2023-10-07

**Authors:** Jessica S. Lemos, Elena Blundo, Antonio Polimeni, Marcos A. Pimenta, Ariete Righi

**Affiliations:** 1 Departamento de Física, Universidade Federal de Minas Gerais, Belo Horizonte 31270-901, MG, Brazil; mpimenta@fisica.ufmg.br; 2 Dipartimento di Fisica, Sapienza Università di Roma, 00185 Roma, Italy; elena.blundo@uniroma1.it (E.B.); antonio.polimeni@uniroma1.it (A.P.); 3 Departamento de Física, Universidade Federal do Ouro Preto, Campus Universitário Morro do Cruzeiro, ICEB, Ouro Preto 35400-000, MG, Brazil

**Keywords:** MoS_2_ dome, Raman excitation profile, biaxial strain

## Abstract

This work describes a resonance Raman study performed in the domes of monolayer MoS_2_ using 23 different laser excitation energies covering the visible and near-infrared (NIR) ranges. The multiple excitation results allowed us to investigate the exciton–phonon interactions of different phonons (A${}^{\prime }$_1_, E${}^{\prime }$, and LA) with different excitonic optical transitions in biaxially strained monolayer MoS${}_{2}$. The analysis of the intensities of the two first-order peaks, A${}^{\prime }$_1_ and E${}^{\prime }$, and the double-resonance 2LA Raman band as a function of the laser excitation furnished the values of the energies of the indirect exciton and the direct excitonic transitions in the strained MoS${}_{2}$ domes. It was noticed that the out-of-plane A${}^{\prime }$_1_ phonon mode is significantly enhanced only by the indirect exciton I and the C exciton, whereas the in-plane E${}^{\prime }$ mode is only enhanced by the C exciton of the MoS_2_ dome, thus revealing the weak interaction of these phonons with the A and B excitons in the strained MoS${}_{2}$ domes. On the other hand, the 2LA Raman band is significantly enhanced at the indirect exciton I and by the A (or B) exciton but not enhanced by the C exciton, thus showing that the LA edge phonons that participate in the double-resonance process in MoS${}_{2}$ have a weak interaction with the C exciton.

## 1. Introduction

Two-dimensional (2D) materials exhibit unique properties that depend on various factors, including the material’s chemical composition, atomic arrangement, thickness, and interlayer interactions [1,2]. Modification of the intrinsic properties of crystalline materials is often necessary to achieve new fundamental effects or create favorable conditions for device fabrication. By carefully tuning these parameters, researchers can unlock new properties and behaviors in solid-state materials, leading to innovative technologies and scientific discoveries [2,3,4]. Recent studies have explored the effects of mechanical deformation and stress on the properties of 2D materials [5,6,7,8,9]. In particular, the ability of 2D materials to be easily strained is of great interest, as strain engineering can significantly impact their electronic and optical properties [8,10,11,12,13,14,15,16]. Indeed, many theoretical works have predicted that biaxial strain is particularly effective in tuning the band structure of transition metal dichalcogenides (TMDs) [17,18,19,20,21,22].

Atomically thin materials of the MoS_2_-type family are particularly well-suited for studying the effects of mechanical deformation and stress, because they can withstand extreme nonhomogeneous deformations before rupture [9,23,24,25,26,27,28,29]. The electronic and optical properties of MoS_2_ monolayers are strongly coupled to the valley/spin/orbital degrees of freedom and the lattice structure, making them sensitive to mechanical deformation or stress [5,11,25,30,31,32]. To explore the effect of biaxial strain on TMDs, various methods have been developed, such as deposition on nanocones [33] or pillars [34] and the epitaxial growth of superlattices [35]. In these methods, the strain obtained is typically around 1–2%. However, values of strain of 5% are reached at the center the of MoS_2_ monolayer domes that are investigated in this work [11,36].

In this paper, we present a resonant Raman spectroscopy study of an MoS_2_ dome sample using 23 different laser excitation energies in the NIR and visible ranges. Our measurements allowed us to obtain the Raman excitation profiles (REPs) of the first-order E${}^{\prime }$ and A${}^{\prime }$_1_ Raman modes, as well as the REP of the second-order 2LA Raman band. Our results show that the three Raman bands are significantly enhanced at the indirect exciton transition at 1.61 eV and by the C exciton at 2.72 eV, but the interaction of the first-order modes with the A and B excitons was shown to be very weak in the strained MoS_2_ dome. Redshifts were also observed in the energies of the A and B excitons, in agreement with previous photoluminescence (PL) results [11,18,21,37], as well as in the energy of the C exciton. In fact, the multiple excitation Raman study in strained MoS${}_{2}$ presented in this work is the first report of the REPs of the first- and second-order bands in strained MoS${}_{2}$ and, as far as we know, the redshift that we observed for the C exciton in MoS${}_{2}$ domes has not yet been reported in the literature. Our work provides important insights into the effects of biaxial strain on the excitonic and optical properties of 2D materials, which could have implications for the development of innovative technologies and scientific discoveries.

## 2. Materials and Methods

**Figure 1:**
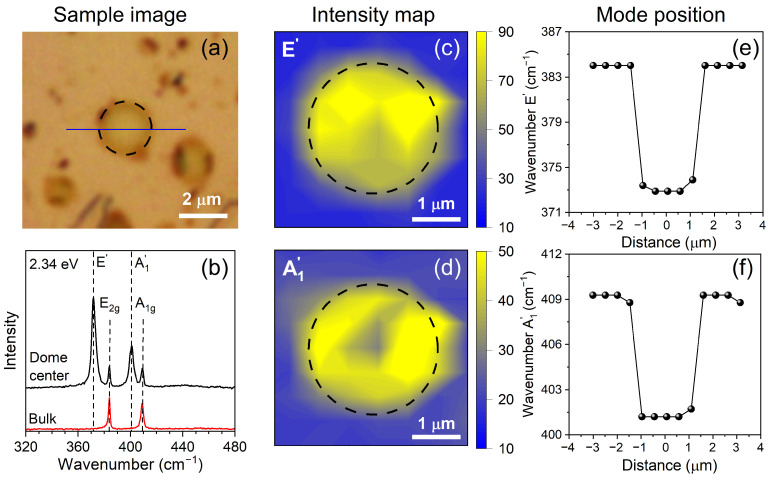
(**a**) Optical image of the sample. The dome edge is delimited by a black dashed line. (**b**) Raman spectra of the dome center (black) and of the MoS_2_ bulk flake (red) acquired with excitation energy of 2.34 eV. (**c**,**d**) Intensity Raman maps of the first-order Raman modes E${}^{\prime }$ and A${}^{\prime }$_1_, respectively. (**e**,**f**) Positions of first-order Raman modes, E${}^{\prime }$ and A${}^{\prime }$_1_, measured along the blue line shown in Figure 1a, respectively.

Figure 1a displays an optical image of an MoS_2_ dome, created according to the procedure outlined by Tedeschi et al. [38] to generate isolates or conglomerates of semispherical nano- and micrometric domes. Raman maps were acquired using a Horiba LabRAM HR Evolution spectrometer equipped with a grating of 1800 lines/mm. A NIKON objective with a magnification of 100× and numerical aperture of 0.9 was used, and the laser energy of 2.34 eV was set to a power of 100 μW to prevent heating and bursting of the dome. In addition to this configuration, another piece of equipment was utilized, the HORIBA T64000 triple monochromator spectrometer, using different laser sources and 23 laser excitation energies, covering a wide range of energies from 1.59 to 2.73 eV. Specifically, the Ar-Kr laser was used for excitation energies from 2.73 to 1.92 eV, and the Ti-Sapphire laser, pumped by a VERDI laser at 532 nm, covered the excitation energy range from 1.69 to 1.59 eV. Two solid-state lasers at 1.88 eV and 2.09 eV and one He-Ne laser at 1.96 eV were also used. An objective of 100× magnification and a laser power of around 10 μW were used to avoid heating and bursting of the dome. Raman measurements were performed with linearly polarized lasers but without an analyzer for the scattered light. All measurements were realized at room temperature with a backscattering configuration.

The photoluminescence measurements were conducted using a HORIBA T64000 triple-monochromator spectrometer in a single mode, which was equipped with a grating of 300 lines/mm. The excitation was carried out with a VERDI laser that has an energy of 2.33 eV. A 100× magnification objective was employed, with the laser power set to approximately 100 μW. These measurements were performed at room temperature, employing a backscattering configuration.

## 3. Results

We began our study by characterizing the Raman modes of a selected dome. Figure 1 presents the optical image, Raman spectra, and intensity Raman maps acquired, as well as the positions of the A${}^{\prime }$_1_ and E${}^{\prime }$ modes from the sample. The dashed circles around the dome delineate its edge. Figure 1a shows that the semispherical dome has a diameter of approximately (2.4±0.1) μm. Figure 1b presents the Raman spectra recorded at the center of the dome (black line) and at the MoS${}_{2}$ bulk substrate (red line) with 2.34 eV laser energy. The bulk’s Raman spectrum exhibits two modes, E${}_{2g}$ and A_1g_, located around 384 cm^−1^ and 409 cm^−1^, respectively [39,40]. The Raman spectra at the dome’s center exhibit four modes, where the two weaker peaks come from the bulk’s substrate and the two more intense peaks, shifted to lower wavenumbers around 373 cm^−1^ and 402 cm^−1^, are associated with the E${}^{\prime }$ and A${}^{\prime }$_1_ modes of the monolayer dome, respectively. In the literature, the positions of the E${}^{\prime }$ and A${}^{\prime }$_1_ modes in unstrained monolayer MoS${}_{2}$ are 385 cm^−1^ and 404 cm^−1^, respectively [40,41,42]. Therefore, the redshifts in the mode positions of the strained monolayer MoS${}_{2}$ studied in this work with respect to the unstrained monolayer MoS${}_{2}$ are 12 cm^−1^ and 2 cm^−1^ for the E${}^{\prime }$ and A${}^{\prime }$_1_ modes, respectively [42]. Previous studies have shown that the effect of the biaxial strain at the dome is more significant for the E${}^{\prime }$ mode, with a displacement rate of 2.2 cm${}^{-1}$/% [5]. According to this relation, the shift of 12 cm${}^{-1}$ observed in our work for the E${}^{\prime }$ mode corresponds to a value of ≈5.5% of strain at the dome’s center, shown in Figure 1a.

Figure 1c–f shows the intensity maps and positions of the dome peaks acquired with a laser energy of 2.34 eV. The intensity maps of the E${}^{\prime }$ and A${}^{\prime }$_1_ Raman peaks indicate an increase in the modes intensities at the dome’s region compared with the bulk spectra, as displayed in Figure 1c,d, respectively. The positions of the E${}^{\prime }$ and A${}^{\prime }$_1_ Raman peaks at the monolayer dome’s center are redshifted by about 11 cm^−1^ and 7 cm^−1^, respectively, when compared with the bulk’s positions, as shown in Figure 1e,f, respectively.

**Figure 2:**
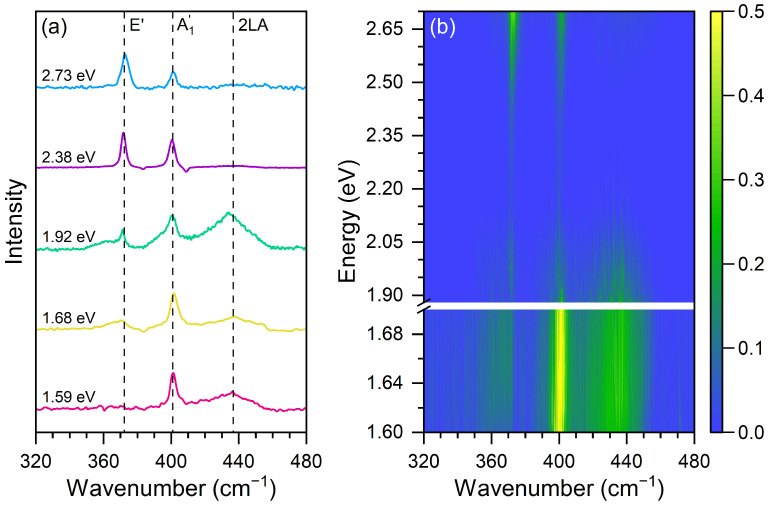
(**a**) Raman spectra of the MoS_2_ dome center with five excitation energies: 1.59 eV (pink line), 1.68 eV (yellow line), 1.92 eV (green line), 2.38 eV (purple line), and 2.73 eV (blue line). (**b**) Raman map of the dome center with twenty-three laser lines with photon energies in the Vis–NIR range.

Figure 2a shows the Raman spectra recorded at the dome’s center using five different laser excitation energies: 1.59 eV (780 nm), 1.68 eV (738 nm), 1.92 eV (646 nm), 2.38 eV (521 nm), and 2.73 eV (454 nm). The three dashed lines serve as guides to follow the positions of the in-plane E${}^{\prime }$, the out-of-plane A${}^{\prime }$_1_, and the 2LA modes. The Raman spectra of the bulk sample were subtracted from the spectra so that it only displays the peaks of the dome’s center. In addition to the first-order modes, we can observe the second-order 2LA Raman band [43] centered around 440 cm^−1^.

In the spectrum of the 1.59 eV excitation energy, we can observe an intense and sharp peak associated with the out-of-plane A${}^{\prime }$_1_ mode and the broad 2LA band. Notice that the in-plane E${}^{\prime }$ mode is absent in this spectrum. In the 1.68 eV excitation energy spectrum, the A${}^{\prime }$_1_ mode is still the most intense, the 2LA band is observed, and we can see the appearance of a broad band in the position of the E${}^{\prime }$ mode. In the 1.92 eV excitation energy spectrum, we can observe that the broad 2LA band becomes the most intense, and the appearance of the E${}^{\prime }$ mode is clearly observed. The broad band near the position of the E${}^{\prime }$ mode is related to a double-resonance Raman process involving different valleys of MoS${}_{2}$ [43]. By increasing the excitation energy, we can observe in the 2.38 eV excitation energy spectrum that the E${}^{\prime }$ mode becomes as intense as the A${}^{\prime }$_1_ mode, and the 2LA band is absent in the spectrum. In the highest 2.73 eV excitation energy spectrum, we see that the E${}^{\prime }$ mode becomes more intense than the A${}^{\prime }$_1_ mode.

Figure 2b shows the result of a multiple excitation energy Raman map at the MoS_2_ dome’s center obtained using 23 different laser lines with excitation energies ranging from 1.59 to 2.73 eV. The peak intensities in Figure 2b were normalized by the intensity of the 465 cm${}^{-1}$ Raman mode of quartz and are represented by the color bar. The intensity of the Raman spectrum of quartz has no dependence on the laser excitation energy since its band gap is in the UV range, around 8 eV [44,45] and, therefore, there is no resonance Raman effect in quartz in the visible range. The energies of the incident photons are on the vertical scale, and the horizontal scale represents the Raman shifts. The blank gap in excitation energies between 1.69 and 1.88 eV in Figure 2b is a region where we do not have available laser lines.

**Figure 3:**
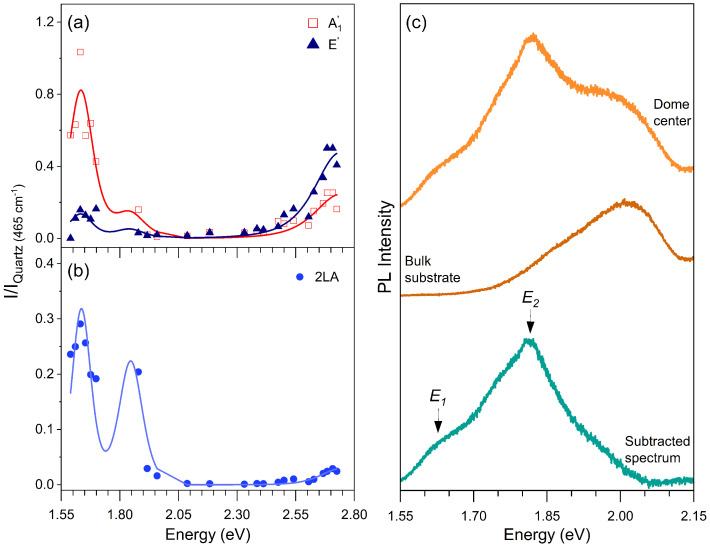
(**a**) Raman excitation profile (REP) of the first-order Raman modes E${}^{\prime }$ (dark blue triangles) and A${}^{\prime }$_1_ (open red squares). (**b**) REP of the 2LA Raman band (blue circles). (**c**) Photoluminescence spectrum acquired at the dome center (orange), MoS${}_{2}$ bulk substrate (brown), and subtracted spectrum (green) resulting from the difference between the dome center and bulk spectra. The arrows indicate the values of the resonance energies obtained in the REP.

Figure 3 shows the Raman excitation profiles (REPs) of the A${}^{\prime }$_1_, E${}^{\prime }$, and 2LA bands, that is, the intensity of each feature as a function of the laser excitation energy. We discuss the results of the first-order modes and the 2LA band separately. The REPs of the first-order A${}^{\prime }$_1_ (open red squares) and E${}^{\prime }$ (dark blue triangles) modes are shown in Figure 3, and the lines represent the best fits of the experimental data considering the equation for Raman intensity as a function of the laser energy *E*_*L*_ for a first-order (one-phonon) process given by [46]

(1) $$I^{k}\left(E_{L}\right)=\sum_{i}{\left|\frac{M_{\mathrm{el}-\mathrm{rad}}\cdot M_{ex-ph}^{\left(i)-(k\right)}\cdot M_{\mathrm{el}-\mathrm{rad}}}{\left(E_{L}-E_{ex}^{i}-i\gamma^{i}\right)\left(E_{L}-E_{ph}^{k}-E_{ex}^{i}-i\gamma^{i}\right)}\right|}^{2},$$

where the index *i* denotes the excitonic transition, the index *k* denotes the two phonons (A${}^{\prime }$_1_ or E${}^{\prime }$), and the three terms in the numerator represent the matrix elements of the electron–radiation (absorption of the incident photon), exciton–phonon, and electron–radiation (emission of the scattered photon) interactions, respectively. The two terms in the denominator give rise to the resonant enhancement of the Raman peaks when the incident or scattered photon energies match the exciton energy. The damping constant $\gamma^{i}$ is related to the finite lifetime of the exciton *i* involved in the Raman process, where *E*${}_{ex}^{i}$ is the energy of exciton *i*, and *E*${}_{ph}^{k}$ is the energy of phonon *k*.

Figure 3a clearly shows three resonances in the REPs in the investigated range of energies. Notice that the out-of-plane A${}^{\prime }$_1_ mode is enhanced by the three resonances, whereas the in-plane E${}^{\prime }$ mode is weakly enhanced at lower energies but is more intense than the A${}^{\prime }$_1_ mode at higher energies in Figure 3a.

In the fitting process, the same set of parameters of the excitons (energies and damping constants) was used to fit the REPs of the different phonon modes. Table 1 shows the fitting parameters (exciton energies and damping constants) that provide the best fit of the experimental REP data and are represented by the solid curves in Figure 3a. The values for the first resonance (*E*${}_{1}$) are more accurate, since we have more experimental data in this energy region. The lack of experimental points in the range 1.69–1.88 eV prevented us from measuring with accuracy the second resonance (*E*${}_{2}$) energy and linewidth. The accuracy in the value of the third resonance energy (*E*${}_{3}$) is also poor, since we do not have experimental points above 2.8 eV.

We now discuss the resonance Raman behavior of the 2LA band that comes from a second-order phonon process involving two phonons of the longitudinal acoustic (LA) branch near the Brillouin zone edge (K and M points) [43]. Figure 3b shows the REP of the 2LA mode, where the blue circles represent the experimental data and the curves represent the best fit considering the expression for the intensity of a second-order Raman process as a function of the laser energy *E*_*L*_ given by the following expression [43]:

(2) $$I^{2LA}\left(E_{L}\right)=\sum_{i}{\left|\frac{M_{\mathrm{el}-\mathrm{rad}}\cdot M_{ex-ph}^{(i)-LA}\cdot M_{ex-ph}^{(i)-LA}\cdot M_{\mathrm{el}-\mathrm{rad}}}{\left(E_{L}-E_{ex}^{i}-2E_{ph}^{LA}-i\gamma^{i}\right)(E_{L}-E_{ex}^{i}-E_{ph}^{LA}-i\gamma^{i})\left(E_{L}-E_{ex}^{i}-i\gamma^{i}\right)}\right|}^{2},$$

where the two middle terms in the numerator of Equation (2) represent the exciton–phonon interactions involving two phonons with opposite momenta. The denominator shows three terms that give the resonances of the incident photon and the scattered photon with one and two phonons. The REP of the 2LA Raman band shown in Figure 3b was fitted using the same energy values of *E*${}_{1}$, *E*${}_{2}$, and *E*${}_{3}$, shown in Table 1. We can observe that the 2LA band is significantly enhanced at lower energies (*E*${}_{1}$ and *E*${}_{2}$) but only very weakly enhanced at the higher energy resonance (*E*${}_{3}$).

Figure 3c shows the photoluminescence spectra recorded at the dome center (upper spectrum), at the MoS${}_{2}$ bulk substrate (middle spectrum), and the subtraction between the two first ones in the bottom, where we can only observe the contribution of the strained MoS${}_{2}$ single layer.

## 4. Discussion

Let us now discuss the physical origin of the three resonances *E*${}_{1}$, *E*${}_{2}$, and *E*${}_{3}$ observed in the resonance Raman results. For the first-order A${}^{\prime }$_1_ and E${}^{\prime }$, we observed that the out-of-plane A${}^{\prime }$_1_ mode is strongly enhanced at *E*${}_{1}$ and *E*${}_{3}$, whereas the in-plane E${}^{\prime }$ is only strongly enhanced at the higher resonance energy *E*${}_{3}$. On the other hand, the second-order 2LA band is significantly enhanced at *E*${}_{1}$ and *E*${}_{2}$ and practically not observed at *E*${}_{3}$.

The highest resonance energy *E*${}_{3}$ can be attributed to exciton C of MoS${}_{2}$, whose energy in unstrained monolayer MoS${}_{2}$ is around 2.9 eV, as observed by resonance Raman spectroscopy [46], and is far isolated from the other lower energy excitons in the optical spectrum. The observed value of the C exciton energy at 2.72 eV allows us to conclude that it is redshifted by about 0.18 eV in the strained domes with respect to the unstrained MoS${}_{2}$ monolayer, similar to the case of the energies of A and B excitons in the strained MoS${}_{2}$ domes. Interestingly, the 2LA band is only very weakly enhanced at higher energies, thus revealing a weak interaction of the zone-edge LA phonons with the C exciton.

In order to assign the *E*${}_{1}$ and *E*${}_{2}$ resonances in the REPs, we acquired the photoluminescence (PL) spectrum on the same dome, whose REP curves are displayed in Figure 3a,b. The topmost trace in Figure 3c is the PL spectrum recorded with the laser spot centred on the top of the MoS${}_{2}$ dome. The emission from the MoS${}_{2}$ substrate (or bulk) adjacent to the dome is given by the middle trace in Figure 3c. Finally, the bottommost trace in Figure 3c is obtained by subtracting the bulk contribution from the spectrum recorded on the dome. It must be emphasized that the spectrum intensities were not corrected by the spectral response of the spectrometer (detector and gratings), and the intensities at lower energies are underestimated. Nevertheless, in the PL spectrum, two clear resonances are observed. They match the *E*${}_{1}$ and *E*${}_{2}$ REP resonances very well, which are indicated by the arrows in the lower part of Figure 3c.

Previous PL studies on strained MoS${}_{2}$ domes similar to those investigated here showed the presence of two main contributions to the emission spectrum acquired close to the top of the dome [11], like in the present case. The lower and higher energy resonances in the PL spectrum were attributed to the indirect and direct excitons, respectively. As a matter of fact, for sufficiently high strain values (typically greater than 2%), the maximum of the valence band (VB) of the MoS${}_{2}$ monolayer undergoes a transition from K to Γ (the same occurs in WS${}_{2}$ and WSe${}_{2}$ monolayers, as reported in ref. [11]). At the same time, the minimum of the conduction band (CB) at K moves at lower energy, while remaining the lowest state of the CB. Consequently, the exciton transition with the lowest energy becomes indirect in character ($\Gamma_{\mathrm{VB}}$ − K${}_{\mathrm{CB}}$), while the direct exciton transition (K${}_{\mathrm{VB}}$ − K${}_{\mathrm{CB}}$) is at higher energy. It should be noted though that when the direct and indirect excitons are resonant, they hybridize, and their direct vs indirect character is smeared out. In particular, the indirect exciton may gain sufficient oscillator strength and become bright despite its k-space indirect character. Recently, evidence of exciton hybridization was also observed in the strain dependence of the exciton magnetic moment in WS${}_{2}$ domes [13]. By comparing the PL difference spectrum in Figure 3c with the results on MoS${}_{2}$ domes reported in ref. [11], we may attribute the *E*${}_{1}$ resonance in the REPs to the indirect exciton and the *E*${}_{2}$ resonance to the direct exciton. Indeed, in ref. [11], it was observed that the indirect exciton energy ranges between 1.65 eV and 1.62 eV depending on the position on the dome, in agreement with the *E*${}_{1}$ resonance. The direct exciton was found to vary from 1.75 eV to 1.78 eV for increasing strain, which suggests the *E*${}_{2}$ resonance (*E*${}_{2}$ = 1.82 ± 0.05 eV) is associated with the direct (or A) exciton state. Nevertheless, due to the large energy uncertainty of *E*${}_{2}$ and its low spectral weight, we cannot exclude a possible contribution from the B exciton. Notice that the energies of the A and B excitons obtained from the REPs of the Raman modes in the unstrained single layer of MoS${}_{2}$ are 1.90 eV and 2.05 eV [46], showing that A and B exciton energies in MoS${}_{2}$ domes are redshifted with respect to the case of unstrained 1L-MoS${}_{2}$.

Finally, the different relative weight of the indirect (corresponding to *E*${}_{1}$) and direct (corresponding to *E*${}_{2}$) excitons in the PL spectra and in the REP curves probably comes from the exciton–phonon interaction. The PL process involves two optical transitions, and the Raman process involves not only the two optical transitions but also the exciton–phonon interaction, whose matrix element is given by the middle term in the numerator of Equation (1), which is specific for each exciton i and phonon k.

## 5. Conclusions

In conclusion, this study presents a resonance Raman analysis of the first-order modes and of the double-resonance 2LA band in a dome of strained monolayer MoS_2_, using 23 different laser excitation energies in the range of 1.59 to 2.73 eV. It was observed that the Raman features are enhanced by three resonance energies, *E*${}_{1}$, *E*${}_{2}$, and *E*${}_{3}$ at, respectively, 1.61 eV, 1.82 eV, and 2.72 eV. It was observed that the out-of-plane A${}^{\prime }$_1_ mode is significantly enhanced by the first and the third resonances (*E*${}_{1}$ and *E*${}_{3}$), while the in-plane E${}^{\prime }$ mode was only significantly enhanced at *E*${}_{3}$. The 2LA band, which comes from a double-resonance Raman process involving two phonons, exhibited a different resonance behavior, since it was strongly enhanced at the two first resonances *E*${}_{1}$ and *E*${}_{2}$ and practically not observed at *E*${}_{3}$. Our analysis allowed us to ascribe the third resonance to the C exciton of MoS${}_{2}$. On the other hand, the high strain values (about 5%) that can be reached in the MoS${}_{2}$ domes led to a direct-to-indirect band gap transition, with the indirect exciton at lower energy than the direct (or A) exciton. As a matter of fact, the comparison between the strain-dependent PL spectra and the the REP curves indicated that the first and second resonances are consistent with the indirect and direct excitons, respectively. Our results show that the exciton-phonon coupling involving the out-of-plane A${}^{\prime }$_1_ and the in-plane E${}^{\prime }$ modes with the A exciton is small in strained MoS${}_{2}$. On the other hand, the enhancement of the first-order modes by the C exciton, especially strong for the E${}^{\prime }$ mode, reveals a strong exciton–phonon coupling in this case. The second-order 2LA band exhibited a different resonance behavior, since it is was significantly enhanced at the lower energy resonances but practically not observed when the excitation energy matched the exciton C, thus revealing the selective exciton–phonon interaction for the double-resonance Raman process of MoS${}_{2}$-type materials. These findings provide valuable insights into the behavior of MoS_2_ under strain and its potential use in developing strain-based devices, and contribute to the existing knowledge of MoS_2_ physical properties.

## Figures and Tables

**Table 1:** Energies and damping constants of the observed resonances in the Raman excitation profile in strained MoS${}_{2}$.

Resonance	Energy (eV)	Damping (eV)
*E*_1_	1.61 ± 0.01	0.09
*E*_2_	1.82 ± 0.05	0.11
*E*_3_	2.72 ± 0.05	0.19

## Data Availability

The data used to support the findings of this study can be made available by the corresponding author upon request.
